# Case Report: Ambergris coprolite and septicemia in a male sperm whale stranded in La Palma (Canary Islands)

**DOI:** 10.3389/fvets.2024.1388276

**Published:** 2024-04-08

**Authors:** Antonio Fernández, Cristian Suárez-Santana, Paula Alonso-Almorox, Francesco Achille Consoli, Zuleima Suárez González, Ignacio Molpeceres-Diego, Claudia Iglesias González, Marta Lorente Hernández, Amaranta Hugo Pérez, José Luis Martín-Barrasa, Laura Iglesias Llorente, Félix M. Medina, Raiden Grandía Guzmán, Diego Llinás Rueda, Manuel Arbelo, Eva Sierra

**Affiliations:** ^1^Veterinary Histology and Pathology, Institute of Animal Health and Food Safety (IUSA), Atlantic Center for Cetacean Research, Marine Mammals Health WOAH col Centre, University of Las Palmas de Gran Canaria, Veterinary School, Las Palmas, Spain; ^2^Canary Islands Stranding Network, Canary Islands Government, Canary Islands, Spain; ^3^Group of Fish Health and Infectious Diseases, University Institute of Animal Health, and Food Safety (IUSA), University of Las Palmas de Gran Canaria, Veterinary School, Las Palmas, Spain; ^4^Country Animal Facility, Research Unit, Hospital Universitario de Gran Canaria Dr. Negrín, Las Palmas de Gran Canaria, Spain; ^5^Microbiology Department, Hospital Universitario de Gran Canaria Dr. Negrín, Las Palmas de Gran Canaria, Spain; ^6^Biodiversity Unit, Cabildo de la Isla de La Palma, Canary Islands, Spain

**Keywords:** Ambergris, sperm whale, stranding, septicemia, pathology

## Abstract

On the 21st of May 2023, a dead adult male sperm whale (*Physeter macrocephalus*) of 13 m in length and estimated weight of around 18,000 kg was reportedly stranded at Playa Los Nogales, La Palma, Canary Islands, Spain. A necropsy was performed 48hpm. A 50 cm diameter and 9.5 kg coprolite was found obstructing the caudal colon-rectal lumen. Necro-hemorrhagic lesions were found in heart muscles and three different bacteria of intestinal origin were isolated and identified (*Edwarsiella tarda*, *Hathewaya limosa* and *Clostridium perfringens*). It is reported a lethal septicemia of intestinal origin associated with ambergris coprolite as cause of death in this sperm whale.

## 1 Introduction

Sperm whales (*Physeter macrocephalus*) are the “kings and queens” around the Canary Islands, a hotspot for cetacean biodiversity in the eastern Atlantic Ocean, with up to 30 other described species that are resident or visitors of these oceanic deep waters (1, 2). The stranding of these sea giants has always captured the public eye with curiosity and has raised great interest in the scientific community, with two main questions persisting throughout time: What makes these animals strand, and what are their causes of death?

There have been less than a hundred reports of sperm whale strandings in this region in the last 50 years. Moreover, in the last 25 years, around 50 sperm whales (from a total report of 1,200 stranded cetaceans) have been subjected to a systematic complete or partial necropsy by veterinarians and veterinary pathologists at the Institute of Animal Health, Atlantic Center for Cetacean Research, (Veterinary School, Universidad Las Palmas de Gran Canaria).

These strandings are particularly significant due to the increasing social awareness and concern around the link of these deaths with anthropogenic activities (3, 4). Geographically, the Canary Islands region holds history in the presence of many cetacean strandings related to anthropogenic activities, such as the proven cause-effect of the use of Mid Frequency Active Sonar (MFAS) by military operations with the mass stranding of beaked whales in the early 2000s, that lead to the implementation of an antisubmarine sonar ban around the archipelago since 2004 (5–7). Similarly, vessel strikes or collisions, as another important cause of death of sperm whales, are currently under the scope of research (8–10). Nevertheless, besides these human-related events, naturally derived causes of death must not be left aside since they also play an essential role in the mortality of these individuals (3, 11). Therefore, in the context of a thorough forensic investigation, determining whether the stranding of a sperm whale in the Canary Islands is linked to human activities or of natural origin, is as essential as it is challenging.

The presence of coprolites in sperm whales has been reported for centuries, not merely as a casualty but because of the significative importance and high value these so-called “Ambergris stones” have had throughout history in the perfumery industry, among others (12). These coprolites are estimated to occur more predominantly in males, with a prevalence of about in 1 out of a 100 sperm whales (13, 14).

Through the present case report, we provide a factual example of such challenging events, by describing a systematic pathological examination and ancillary laboratory analyses of a sperm whale stranding, with a final diagnosis of a lethal septicemia from intestinal origin linked to a colonic obstruction caused by a 9.5 kg coprolite (ambergris stone).

## 2 Case description

**Figure 1:**
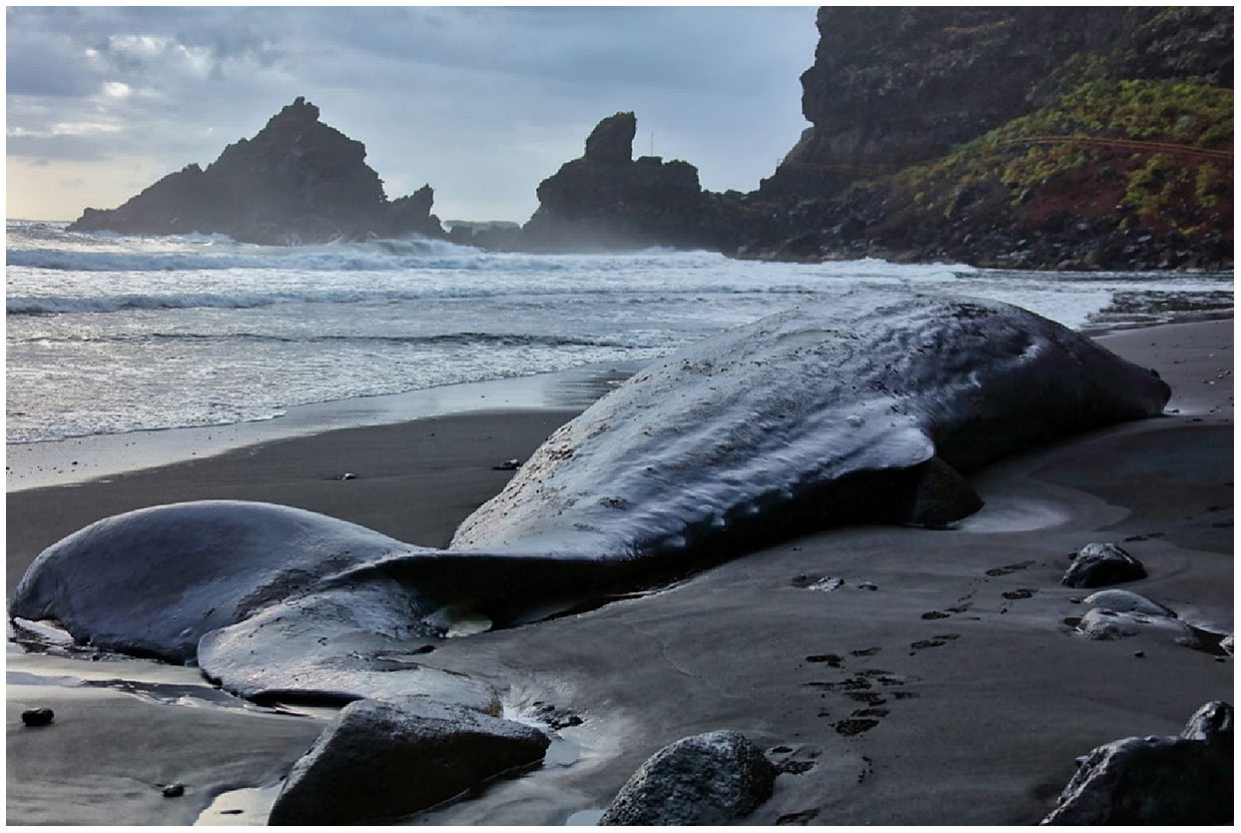
Stranded sperm whale at Playa Los Nogales.

On the 21st of May 2023, a dead adult male sperm whale (*Physeter macrocephalus*) of 13 m in length and estimated weight of around 18,000 kg was reportedly stranded at Playa Los Nogales, La Palma, Canary Islands, Spain. The animal showed no external injuries and a very fresh condition, a postmortem interval of 12 h was established according to images and video taken on that morning (Figure 1).

The following day, on the 22nd of May, the corresponding authorities tried to tow the carcass through the sea to an authorized location for necropsy, but due to the problematic geographical characteristics of the area and several logistical impediments for the operation, the Canary Islands Stranding Network team was then mobilized for an *in situ* necropsy on the 23rd of May (48 h after the stranding report). This team involved Veterinary Pathology specialists from the Institute of Animal Health (IUSA) at Universidad de Las Palmas Gran Canaria (ULPGC).

The ambient temperature ranged from 14°C to 24°C at the stranding location (Los Nogales beach, La Palma), and the carcass was partially covered by water during high tides. Nevertheless, the animal showed signs of rapid decomposition, establishing a decomposition code at the time of necropsy of 3–4 (15) following a 1–5 scale (16). The stranded carcass had beached on its right side but had been dragged by the tide to a final left-sided position. Therefore, the opening of the thoracic and abdominal cavities was performed on the right side.

During the external gross examination, dilation of the abdominal area, with rupture of the skin, subcutaneous layers, and muscles in the caudal dorsal area from which intestinal loops protruded was noted. No other external lesions were identified.

Few parasitic cysts (*Phylobothrium* spp.) were detected within the blubber after dissection of the dorsal skin. Muscles of the dorsal thoracic and abdominal areas showed focal extensive multifocal hemorrhagic.

In the thoracic cavity, the lungs were bilaterally congested and partially collapsed, with pink to red pleural surfaces showing multifocal hemorrhagic areas with associated material compatible with fibrin. Internally, after cutting through the cranial, medial, and caudal lobule locations, alveolar edema and blood were present in the lung parenchyma and within the larger bronchi, but no areas resembling pneumonia or bronchopneumonia were identified. Tracheal lymph nodes were edematous but normal in size.

**Figure 2:**
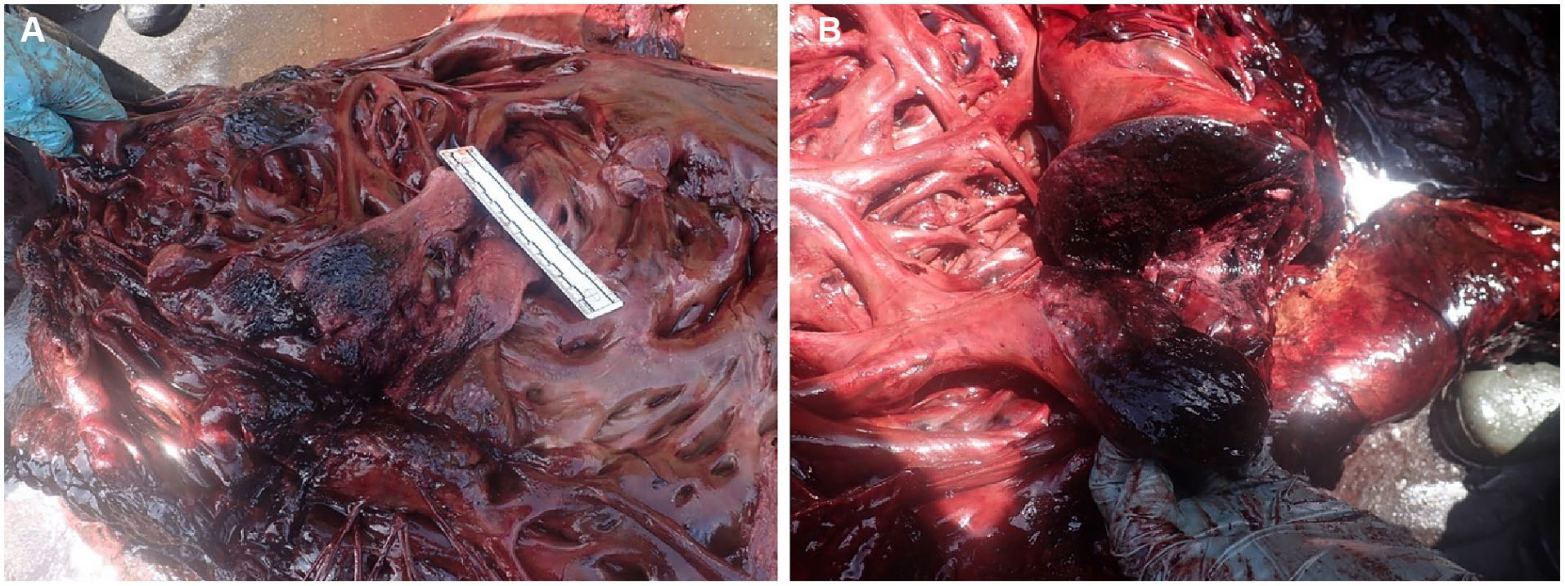
**(A)** Epicardial and myocardial necro-hemorrhagic areas in the heart. **(B)** Local extensive hemorrhages within the heart papillary valve muscle in the left ventricle.

One of the most visible gross pathological findings was located in the left side of the ventricular heart endocardium, where marked multifocal hemorrhagic areas could be easily distinguished from the non-hemorrhagic tissue. These hemorrhagic areas extended locally from the epicardial surface deep into the myocardial muscles (Figure 2A). These findings were especially notable in the papillary muscles of the left ventricle (Figure 2B), and not so evident by gross examination in the myocardium of the right ventricle.

No thrombi or major clots were identified inside of the ventricular cavities, neither within the aorta or pulmonary veins. Blood was watery to viscous within the thoracic and abdominal aorta, pulmonary veins, and mesenteric veins.

After opening the very distended abdominal cavity, an enlarged black congestive liver was observed, with a very friable consistency and oozing black fluid from its parenchyma when cutting through. Kidneys were also very soft and friable.

The different stomachal compartments were markedly dilated by gas. The gastric content was scant, with some squid beaks and fish lenses as part of the collected content. A 5 cm metal fishing hook was also found among the gastric content.

**Figure 3:**
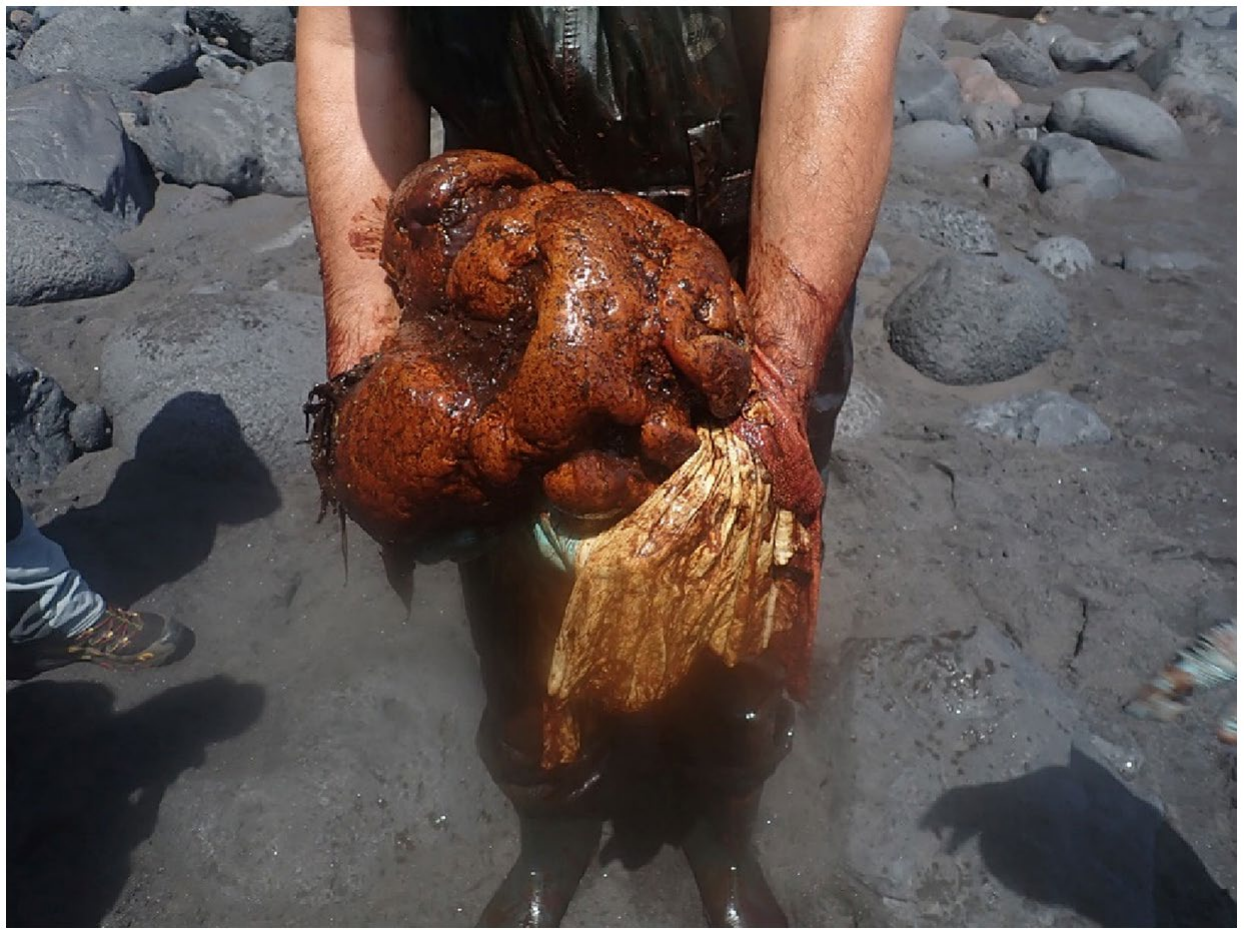
Coprolite found in the distal colon-rectal intestine of the sperm whale: The white-yellowish membrane detached from the surface is also present in the picture.

The distal part of the gastrointestinal tract was extremely dilated with scarce content and no presence of parasites. Within this colon-rectal lumen (1 to 2 meters from the anus), a putrid smelling 9.5 kg solid coprolite with irregular external morphology and 50 cm diameter (Figure 3), was found obstructing the intestinal lumen. A thick yellow membrane was attached to the surface of the coprolite, and parts of squid beaks were observed to be embedded and visible on the surface of this coprolite.

Tissues of all major organs and observed lesions were collected and stored in either neutral buffered 10% formalin fixative solutions for histology or directly frozen at −80°C for microbiological analysis.

After fixation in buffered formalin, histological samples were trimmed and processed, embedded in paraffin, sectioned at 5 μm thickness, and stained with hematoxylin and eosin for light microscopy examination. Additional histochemical techniques were carried out (Gram and Masson’s Trichrome stains) to better evaluate tissues.

**Figure 4:**
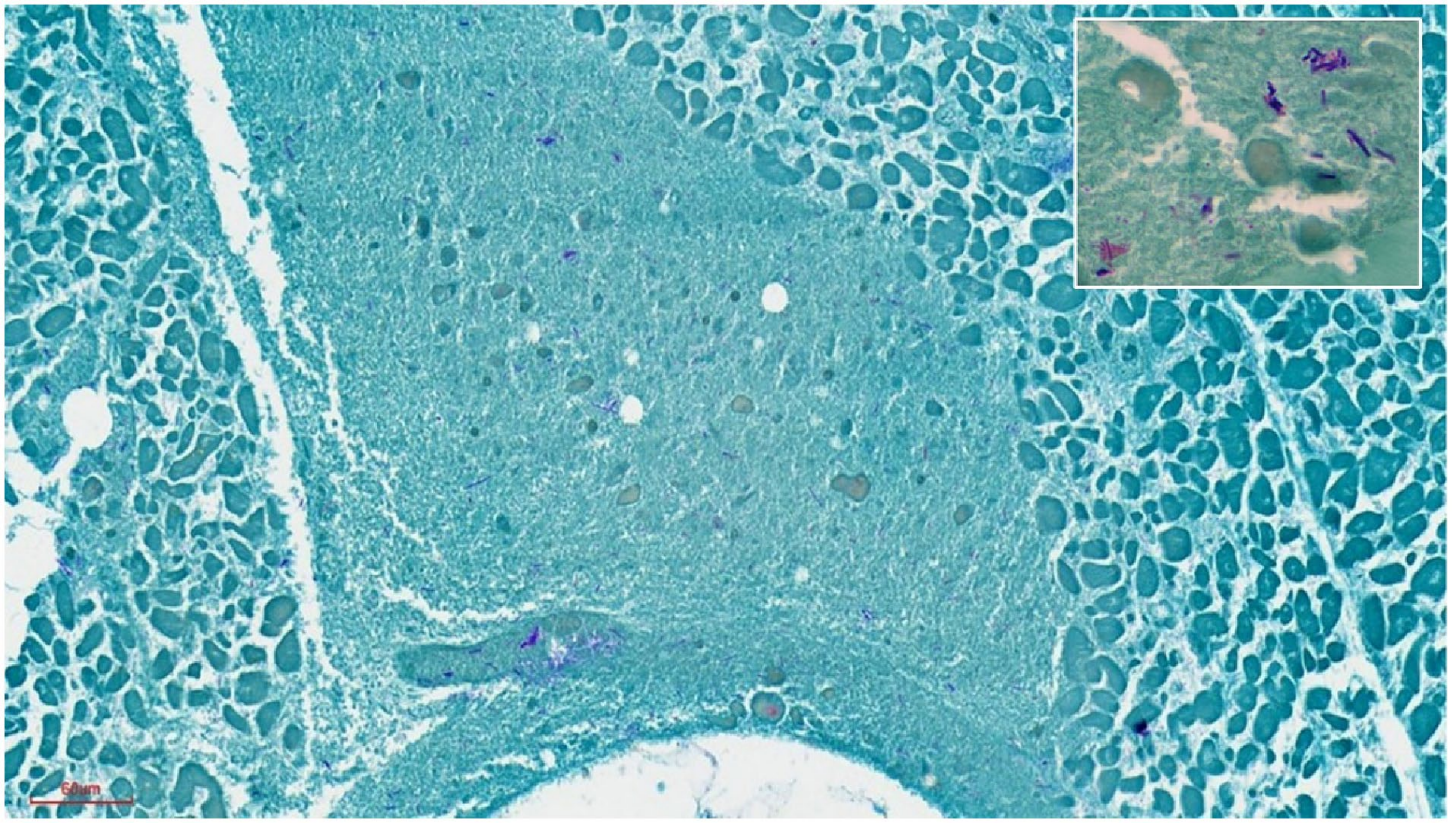
Heart muscle. Severe extensive myo-cytolysis with abundant Gram-positive and Gram-negative bacteria in the myocardium. 20x. Inset: gram + and – bacteria. Gram staining. 100X.

Histologically, all organs showed a moderate to advanced autolysis state and morphology. The lesions were consistent with the gross examination findings. Necro-hemorrhagic necrosis in myocardium and skeletal muscle associated with Gram + and Gram - coco-bacillary bacteria were revealed by histochemical techniques (Figure 4). These were also present systemically in other organs (e.g.: lungs, liver, skeletal muscle, intestine, etc.).

The intestinal mucosa of the distal colon was very autolytic with significant presence of bacteria. The membranous tissue that covered the coprolite showed a non-glandular hyperkeratotic stratified epithelium (squamous metaplasia).

For microbiological culturing, samples were processed within 24 h of collection. To investigate aerobic and anaerobic bacteremia, 3 mL of blood were inoculated on 10 mL of BD BACTEC Lytic Anaerobic (Becton Dickinson) and incubated in BD BACTEC FX blood culture system (Becton Dickinson) for 5 days. If any sign of growth was detected, a subculture was made on Blood Chocolate and McConkey agar, and plates were incubated for 48 h at 37°C in 5% CO2 atmosphere and on Brucella Blood Agar with Hemin and Vitamin K1 incubated for 4 days at 37°C anaerobically (<0.1% O_2_, >15% CO_2_) (GENbox anaer, bioMérieux SA, Marcy-l’Etoile, France). For the middle intestine, distal intestine (colon/rectum), lung, muscle, heart and liver samples, suspensions were made by vortexing the tissues in 0.9% saline solution, after which 30 μL of the suspensions were plated onto McConkey, Mannitol Salt agar, tryptic soy agar +5% sheep blood, Blood Chocolate agar, and Sabouraud Dextrose Agar, and incubated for 24 h at 37°C, aerobically. To investigate anaerobic microorganisms, 30 μL of the same suspensions were plated onto Brucella Blood Agar with Hemin and Vitamin K1, and Blood Chocolate agar, incubated for 4 days at 37°C anaerobically (<0.1% O_2_, >15% CO_2_) (GENbox anaer, bioMérieux SA, Marcy-l’Etoile, France). All media were from Becton Dickinson GmbH (Heidelberg, Germany). Bacterial species were identified using colony morphology and Gram stain.

For advanced identification the recovered isolates were then identified by matrix-assisted laser desorption ionization time of flight mass spectrometry (MALDI-TOF MS) analysis (MALDI Biotyper®, Bruker Daltonics GmbH & Co.KG, Bremen, Germany). For MALDI-TOF MS identification, fresh colonies were used. Bacterial colonies were directly applied from the culture to the MS plate and left to dry, adding then 1 μL of Bruker Matrix HCCA (Bruker Daltonics GmbH & Co.KG, Bremen, Germany). The identification was based on the score values released by the equipment’s instructions. According to Bruker biotyper’s guidelines, a score value ≥2 was interpreted as high-confidence identifications, which means reliable identification at the species level.

Microbiological analysis identified the presence of *Edwarsiella tarda* in isolates from middle intestine, distal intestine (colon/rectum), heart, blood, lungs, liver, and muscle cultures. *Hathewaya limosa* (formerly known as *Clostridium limosum*) was also identified in middle intestine, heart, blood, lungs, muscle, and liver samples; and *Clostridium perfringens* was identified in distal intestine, heart and blood. The total number of bacterial species isolated from the different samples are shown in Table 1.

**Table 1:** Bacterial strains isolated from sperm whale tissue samples.

	Middle intestine	Distal intestine (colon/rectum)	Blood	Heart	Lung	Muscle	Liver
*Edwarsiella tarda*	+ (2.30)	+ (2.35)	+ (2.33)	+ (2.31)	+ (2.26)	+ (2.38)	+ (2.38)
*Hathewaya limosa*	+ (2.33)	–	+ (2.25)	+ (2.24)	+ (2.21)	+ (2.21)	+ (2.21)
*Clostridium perfringens*	–	+ (2.28)	+ (2.19)	+ (2.14)	–	–	–
*Clostridium butyricum*	–	–	–	*+* (2.29)	–	–	*–*
*Paeniclostridium sordellii*	+ (2.18)	+ (2.21)	–	–	–	–	–
*Enterococcus faecalis*	+ (2.36)	+ (2.42)	+ (2.30)	–	–	–	–
*Proteus vulgaris*	+ (2.54)	+ (2.53)	–	–	–	–	–
*Acinetobacter iwoffi*	–	–	–	–	–	+ (2.38)	–
*Acinetobacter ursingii*	–	–	–	+ (2.26)	–	–	–

(MSV) MALDI-TOF MS score value.

## 3 Discussion

These results indicate the stranded sperm whale was undergoing a systemic bacterial infection of gastrointestinal origin, derived from a “toxic megacolon.” This “toxic megacolon” originated from a chronic active progressive obstructed colon caused by the coprolite. The septicemic bacteria involved, as identified by MALDI-TOF MS analysis were *H. limosa, E. tarda and C. perfringens*; all of which are common bacteria of the gastrointestinal tract that have been associated to cause enteric septicemia (17) - in some cases specifically in marine mammals (18–20); as well as multiorgan hemorrhages and necrosis (21, 22).

*Clostridium limosum* is a Gram-positive anaerobic bacterium, found in the gastrointestinal tract of different species among other environments (23). On the other hand, *Edwarsiella tarda* is a Gram-negative anaerobic bacteria (24), commonly described in numerous aquatic animals (25), that under some circumstances can cause intestinal and extra-intestinal diseases, in fishes, birds, amphibians, mammals (26–30) as well as in marine mammals like wedded seals, and one sperm whale (18). It primarily causes enteric septicemia in fish, humans, and other animals (31), emphysematous putrefactive disease (32), or abnormal swimming behaviors in fish (33), among other alterations and lesions (34, 35).

To our knowledge, this is the second report of sepsis in a sperm whale associated with *E. tarda* as the primary pathogen in the septicemic process (18). In this report, we documented a complete or partially obstructive chronic colonic pathology resulting from the presence of a 50 cm in diameter coprolite. No detailed gastrointestinal tract findings were reported in the case described by Cools (18).

The presence of coprolites in sperm whales has been reported for centuries, not merely as a casualty but because of the significative importance and high value these so-called “Ambergris stones” have had throughout history in the perfumery industry, among others (12).

These coprolites are estimated to occur more predominantly in males, with a prevalence of about in 1 out of a 100 sperm whales (13, 14). The ambergris coprolites are formed as indigestible material like chitinous cephalopods beaks or pens tangle in the intestine, creating a mass that is pushed toward its caudal portion. Here water absorption is maximized (36) and the indigestible material precipitates forming a smooth concretion that does not completely obstruct the intestinal lumen, letting the liquid feces pass (13). As more indigestible fecal material arrives, the coprolite grows. In time, the intestinal wall proximal to the coprolite can distend and eventually either break or cause what we have defined in this case as a pathological “enterotoxic megacolon.”

Toxic megacolon (TM) has been defined in human medicine as a complication of chronic colon diseases, and it must be accompanied by segmental or total distension of the colon and sepsis (37). Among the main etiological factors for TM in humans, inflammatory bowel diseases and bacterial infectious colitis have been described, with Clostridium sp. as one of the primary pathogens (38). In the presented case, a chronic colonic problem and entero-septicemia by *E.tarda*, *C.limosum* and *C. perfringens* were pathologically and microbiological identified.

This report does not only shed light on the potential consequences these coprolites may entail and in the pathogenesis of toxic megacolon-like processes, with significant implications on the health of these species; but it also adds further explanations on the reason why in many cases these ambergris “stones” are found floating in the sea. This would indeed happen when the animal is still able to eject them, and they are not obstructed in the distal colon. Moreover, this study showcases the importance of a complete, systematical post-mortem investigation by competent professionals, even in challenging conditions, as a means for understanding the specific pathologies in different cetacean species.

Although the reasons why only a few male sperm whales do form this kind of stones remains unknown, this thorough veterinary pathological investigation could confirm Herman Melville’s both fictitious and real-life literary masterpiece (39), in which he states: “Who would think, then, that such fine ladies and gentlemen should regale themselves with an essence found in the inglorious bowels of a sick whale! Yet so it is. By some, ambergris is supposed to be the cause, and by others the effect of the dyspepsia in the whale.”

## Data availability statement

The original contributions presented in the study are included in the article/Supplementary material, further inquiries can be directed to the corresponding authors.

## Ethics statement

Ethical approval was not required for the study involving animals in accordance with the local legislation and institutional requirements because the permits and requests are under Canary Islands government. It is a professional work on stranded dead cetaceans with the main aim of diagnosis of cause of death.

## Author contributions

AF: Writing – original draft, Writing – review & editing, Conceptualization, Investigation, Methodology, Supervision. CS-S: Writing – review & editing, Conceptualization, Investigation, Methodology, Resources. PA-A: Investigation, Methodology, Resources, Writing – review & editing. FC: Investigation, Methodology, Resources, Writing – review & editing. ZS: Investigation, Methodology, Resources, Writing – review & editing. IM-D: Investigation, Methodology, Resources, Writing – review & editing. CI: Investigation, Methodology, Resources, Writing – review & editing. ML: Resources, Writing – review & editing. AH: Resources, Writing – review & editing. JM-B: Investigation, Methodology, Resources, Writing – review & editing. LI: Investigation, Methodology, Resources, Writing – review & editing. FM: Resources, Writing – review & editing. RG: Investigation, Methodology, Resources, Writing – review & editing. DL: Visualization, Writing – review & editing. MA: Investigation, Methodology, Resources, Writing – review & editing. ES: Investigation, Methodology, Resources, Software, Writing – review & editing.
