# Supplementary figures and images for: Case Report: Ambergris coprolite and septicemia in a male sperm whale stranded in La Palma (Canary Islands)
Source: Front Vet Sci. 2024 Apr 8;11:1388276. doi: 10.3389/fvets.2024.1388276 (PMC11034612; doi:10.3389/fvets.2024.1388276)

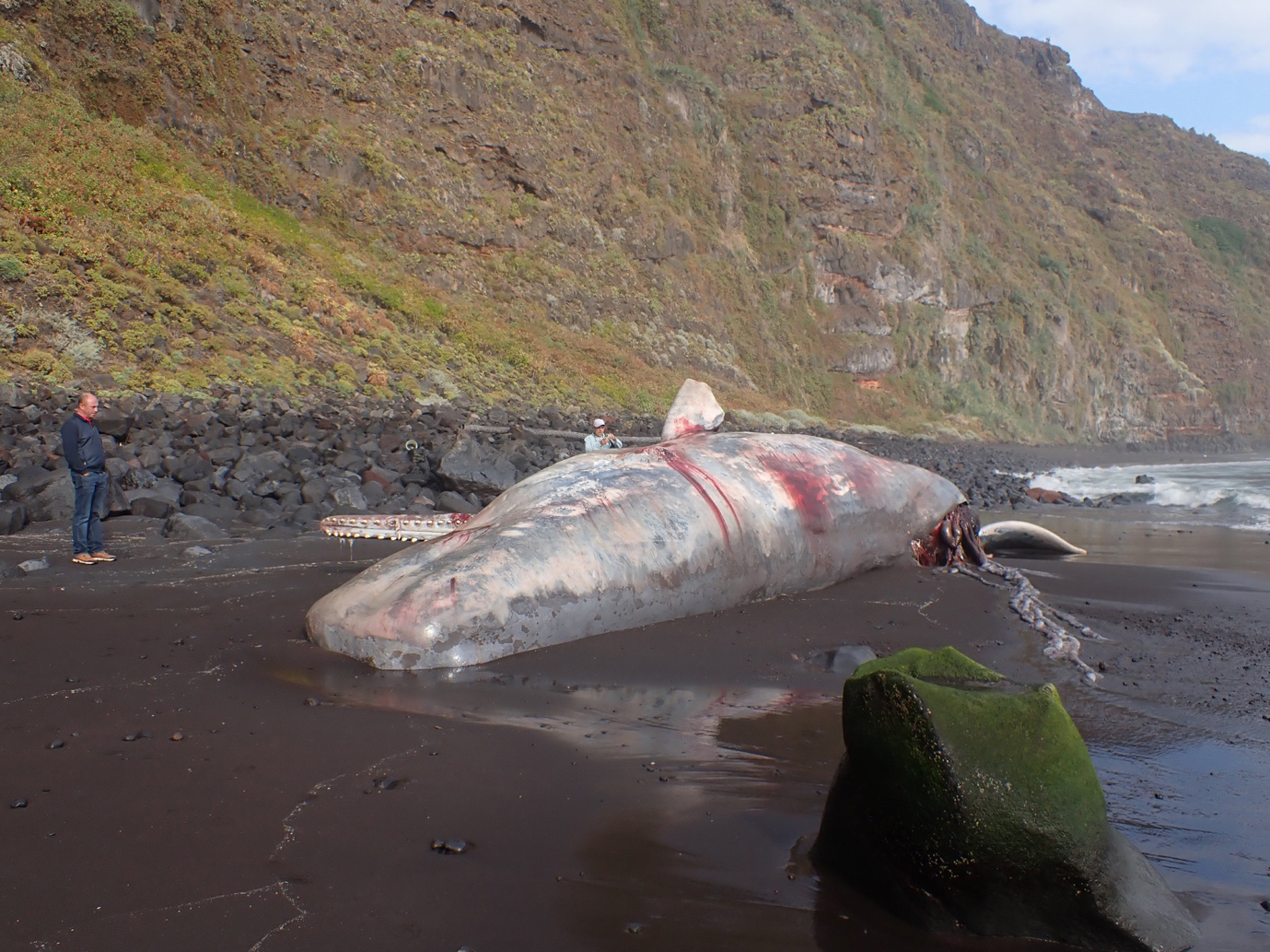

Supplement: Supplementary file 1 [file Image_1.JPEG]
